# Supplementary material for: Demographic and professional risk factors of SARS-CoV-2 infections among physicians in low- and middle-income settings: Findings from a representative survey in two Brazilian states
Source: PLOS Glob Public Health. 2022 Oct 14;2(10):e0000656. doi: 10.1371/journal.pgph.0000656 (PMC10021204; doi:10.1371/journal.pgph.0000656)
Supplement: S1 Text — (DOCX) [file pgph.0000656.s001.docx]

**S1 Text <Sampling equations for sampling replacement>**

Equation 1: $n=\frac{{z_{\alpha}}^{2}*(p*q)}{d^{2}}=\frac{{1.96}^{2}*(0.5*0.5)}{{0.05}^{2}}$

n = sample size

zα = critical value for the standard deviation (1.96)

p = expected likelihood for the variable of interest within the population

q = adjustment for the expected prevalence of the variable of interest

d = sample error

Equation 2: $n_{corr}\frac{N-n}{N-1}*n$

n = sample size

N = physician population in the State
