# Supplementary material for: Demographic and professional risk factors of SARS-CoV-2 infections among physicians in low- and middle-income settings: Findings from a representative survey in two Brazilian states
Source: PLOS Glob Public Health. 2022 Oct 14;2(10):e0000656. doi: 10.1371/journal.pgph.0000656 (PMC10021204; doi:10.1371/journal.pgph.0000656)
Supplement: S1 Table — (DOCX) [file pgph.0000656.s003.docx]

**S1 Table <Multivariate regression models for physicians personal and professional characteristics and probability of being infected with COVID-19>**

| **Maranhão Model (n=551)** |  |  |  |  |
| --- | --- | --- | --- | --- |
| Gender Female | -0.242 | 0.194 | 0.78(0.53-1.15) | 0.213 |
| **Age** |  |  |  |  |
| *> 50 years* |  |  | Ref. | Ref. |
| ***35 to 50 years*** | 0.816 | 0.256 | 2.26(1.36-3.73) | 0.001 |
| ***< 35 years*** | 0.707 | 0.236 | 2.03(1.27-3.22) | 0.003 |
| **Type of Work/Workplace** |  |  |  |  |
| *Office, outpatient or clinic* | -0.109 | 0.265 | 0.89(0.53-1.50) | 0.681 |
| *Diagnostic test equipamento* | -0.638 | 0.199 | 0.53(0.36-0.78) | 0.001 |
| *Surgery (under hospitalization)* | 0.332 | 0.219 | 1.39(0.90-2.14) | 0.129 |
| *Outpatient surgery* | -0.142 | 0.209 | 0.86(0.57-1.30) | 0.499 |
| *Administrative* | -0.647 | 0.214 | 0.52(0.34-0.79) | 0.002 |
| *Teaching and research* | -0.212 | 0.220 | 1.80(0.52-1.24) | 0.334 |
| **Type od practice** |  |  |  |  |
| *Private* |  |  | Ref. |  |
| *Public* | 0.117 | 0.482 | 1.12(0.43-2.89) | 0.808 |
| *Dual-pactice* | 0.118 | 0.472 | 1.12(0.44-2.84) | 0.802 |
| **Workplace in Covid-19 Working** |  |  |  |  |
| *Ward or ICU Covid-19* | 0.316 | 0.201 | 1.37(0.94-2.03) | 0.117 |
| *Non hospitalized cases Covid-19 care* | -0.301 | 0.206 | 0.74(0.49-1.10) | 0.143 |
| *Telemedicine or distance-based consultation in Covid-19* | -0.150 | 0.257 | 0.86(0.52-1.42) | 0.559 |
| *Research in Covid-19* | 0.162 | 0.489 | 1.17(0.45-3.06) | 0.741 |
| *Surveillance or Committees Covid* | 0.178 | 0.448 | 1.19(0.49-2.87) | 0.692 |

| **São Paulo Model (n=632)** |  |  |  |  |
| --- | --- | --- | --- | --- |
| Gender Female | -0.038 | 0.204 | 0.96(0.64-1.43) | 0.854 |
| **Age** |  |  |  |  |
| *> 50 years* |  |  | Ref | Ref. |
| ***35 to 50 years*** | 0.633 | 0.269 | 1.88(1.11-3.19) | 0.019 |
| *< 35 years* | 0.464 | 0.269 | 1.59(0.93-2.69) | 0.084 |
| **Type of Work/Workplace** |  |  |  |  |
| *Office, outpatient or clinic* | -0.337 | 0.244 | 0.71(0.44-1.15) | 0.168 |
| *Diagnostic test equipamento* | -0.025 | 0.226 | 0.97(0.62-1.52) | 0.910 |
| *Surgery (under hospitalization)* | 0.210 | 0.228 | 1.23(0.78-1.92) | 0.357 |
| *Outpatient surgery* | 0.247 | 0.239 | 1.28(0.80-2.04) | 0.300 |
| *Administrative position* | -0.465 | 0.251 | 0.62(0.38-1.02) | 0.063 |
| *Teaching and research* | -0.648 | 0.255 | 0.52(0.38-0.86) | 0.011 |
| **Type od practice** |  |  |  |  |
| *Private* |  |  | Ref. | Ref. |
| *Public* | 0.226 | 0.335 | 1.25(0.65-2.41) | 0.500 |
| *Dual-pactice* | -0.049 | 0.292 | 0.95(0.53-1.68) | 0.867 |
| **Workplace in Covid-19 Working** |  |  |  |  |
| *Ward or ICU Covid-19* | 0.811 | 0.222 | 2.25(1.45-3.47) | <0.001 |
| *Non hospitalized cases Covid-19 care* | -0.156 | 0.226 | 0.85(0.54-1.33) | 0.489 |
| *Telemedicine or distance-basedconsultation in Covid-19* | 0.448 | 0.329 | 1.56(0.82-2.98) | 0.174 |
| *Research in Covid-19* | -0.525 | 0.412 | 1.59(0.26-1.32) | 0.202 |
| *Surveillance or Committees Covid* | -0.118 | 0.505 | 0.88(0.33-2.39) | 0.815 |
