# Supplementary material for: Demographic and professional risk factors of SARS-CoV-2 infections among physicians in low- and middle-income settings: Findings from a representative survey in two Brazilian states
Source: PLOS Glob Public Health. 2022 Oct 14;2(10):e0000656. doi: 10.1371/journal.pgph.0000656 (PMC10021204; doi:10.1371/journal.pgph.0000656)
Supplement: Attachment — Submitted filename: List of changes_3008.docx [file pgph.0000656.s005.docx]

Dear Oliver Ian Tolentino,

In response to your requests to further anonymise the supplementary information and provide guarantees that figures are not protected by copyrights, we have now introduced the following changes.

In the anonymized survey database V2 we have now erased the following information: (a) date of collection; (b) date and time of interview; (c) location data. We believe the database is now completely anonymized, and all the data shared is in accordance with the participant consent.

As per Fig.1, please note that the map is our own original elaboration, and no part of the figure has been previously copyrighted. To construct this figure, we used the IBGE administrative map of Brazil (the base layer) on which we plotted our own survey data. Please note that the IBGE maps of Brazil are NOT COPYRIGHTED, AND ARE COMPLETELY FREE TO USE – these are just BASE LAYERS of the Brazilian administrative territory, that are made available by IBGE for public use, and routinely used by academics for GIS plots. IBGE is a public statistics institute whose function is exactly that of making this type of information publicly available free of charge. Please check directly IBGE’s website to confirm this < <https://www.ibge.gov.br/geociencias/organizacao-do-territorio/malhas-territoriais/15774-malhas.html?=&t=downloads>>.

We understand your journal’s preoccupation with copyrights and with the legality of the material you publish, but in this case, please consider you are really dealing with an original figure, including the base layer of an ordinary map of Brazil made explicitly available by a public institution for research purposes.

We are available to directly discuss this issue further, should you feel the need to.
